# The relationship between hip abductor muscle strength and iliotibial band tightness in individuals with low back pain

**DOI:** 10.1186/1746-1340-18-1

**Published:** 2010-01-13

**Authors:** Amir M Arab, Mohammad R Nourbakhsh

**Affiliations:** 1 Department of Physical Therapy, University of Social Welfare and Rehabilitation Sciences, Evin, Tehran, Iran; 2 Department of Physical Therapy, North Georgia College and State University, Dahlonega, GA, USA

## Abstract

**Background:**

Shortening of the iliotibial band (ITB) has been considered to be associated with low back pain (LBP). It is theorized that ITB tightness in individuals with LBP is a compensatory mechanism following hip abductor muscle weakness. However, no study has clinically examined this theory. The purpose of this study was to investigate the muscle imbalance of hip abductor muscle weakness and ITB tightness in subjects with LBP.

**Methods:**

A total of 300 subjects with and without LBP between the ages of 20 and 60 participated in this cross-sectional study. Subjects were categorized in three groups: LBP with ITB tightness (n = 100), LBP without ITB tightness (n = 100) and no LBP (n = 100). Hip abductor muscle strength was measured in all subjects.

**Results:**

Analysis of Covariance (ANCOVA) with the body mass index (BMI) as the covariate revealed significant difference in hip abductor strength between three groups (P < 0.001). Post hoc analysis showed no significant difference in hip abductor muscle strength between the LBP subjects with and without ITB tightness (P = 0.59). However, subjects with no LBP had significantly stronger hip abductor muscle strength compared to subjects with LBP with ITB tightness (P < 0.001) and those with LBP without ITB tightness (P < 0.001).

**Conclusion:**

The relationship between ITB tightness and hip abductor weakness in patients with LBP is not supported as assumed in theory. More clinical studies are needed to assess the theory of muscle imbalance of hip abductor weakness and ITB tightness in LBP.

## Background

Shortening of the iliotibial band (ITB) has been considered to be associated with low back pain (LBP) [1-4]. Stretching of the ITB is frequently recommended in LBP treatment programs [1,3,5]. However, the exact cause of ITB shortness in persons with LBP has not yet been determined. Anatomically, the ITB is a continuation of the tendinous portion of the tensor fascia lata (TFL) muscle with some contributions from the gluteal muscles. TFL/ITB is a synergist of gluteus medius muscle in hip abduction [6]. Hip abductor muscles play a significant role in control of rotational alignment of the limb and maintaining pelvic lateral stability in single leg stance [1,6,7]. Gottschalk et al [8] believe that the primary function of hip abductors is to stabilize the femoral head in the acetabulum during different parts of the gait cycle. The anterior and middle parts of the gluteus medius have a more vertical pull and help initiate abduction, which is then completed by the TFL/ITB. It is critical that these muscles fire properly through the support phase of the gait cycle, as they eccentrically lengthen while helping to stabilize the pelvis and control femoral adduction in the transverse plane [8].

It is theorized that weakness of hip abductor may cause a compensatory dynamic valgus knee alignment resulting in increased stress on the ITB and consequently ITB shortness [7,9].

Jull and Janda have hypothesized a common muscle imbalance pattern of weakness in gluteus medius and tightness of ITB in chronic musculoskeletal pain syndromes in the lumbar-pelvic-hip area such as chronic LBP [10-12]. Investigators categorized muscles, based on their primary functions, as "phasic" or "postural", and indicated that in response to dysfunction or overuse, the phasic muscles tend to be inhibited or weakened; while the postural muscles tend to develop higher tone and ultimately shorten [10-15]. In this classification, the gluteus medius; primary muscle for hip abduction, is categorized as phasic and TFL/ITB; the synergist muscle, is categorized as postural muscle.

It is assumed that when primary muscle responsible for a specific joint movement is weakened, the synergistic muscle is substituted and become overactive to be the primary muscle responsible for that movement [10,15,16]. Based on these assumptions, it is speculated that ITB shortness in patients with LBP is a compensatory mechanism following hip abductor weakness.

To our knowledge, no study has clinically examined the theory of muscle imbalance of hip abductor weakness and ITB tightness in patients with LBP.

However, some studies have examined the relationship between hip abductor strength and ITB syndrome in runners. With the use of different designs and testing procedures, controversial results have been reported in the studies. Fredericson et al [17] examined hip abductor strength in distance runners with ITB syndrome and a control group of healthy distance runners and found that distance runners with ITB disorder have weaker hip abduction strength compared with healthy subjects. MacMahon and colleagues [18] in a study of 50 runners in which they prospectively evaluated peak hip adduction moments at the beginning of the training programs, found that 7 of the runners subsequently developed ITB disorders and all of whom had significant increased peak hip adduction moments (representative of the decreased ability of the hip abductors to eccentrically control adduction) when compared with non-injured runners. Thus, strengthening of the hip abductors has been recommended for symptom improvement in subjects with ITB dysfunction [17]. In contrast, Grau et al [19] compared the hip abductor strength in 10 healthy runners and 10 runners with ITB syndrome and concluded that weakness of hip abductors does not seem to play a role in the etiology of ITB syndrome in runners.

Some reports have also demonstrated an association between LBP and hip abductor muscle weakness [20-22].

Considering the literature, it seems that the relationship between hip abductor muscle weakness and ITB tightness in patients with chronic LBP warrants further research. The purpose of this study was to evaluate the muscle imbalance of hip abductor weakness and ITB tightness in LBP by investigating the relationship between tightness of ITB and hip abductor muscle strength in subjects with LBP.

## Methods

### Subjects

A total of 300 subjects with and without LBP between the ages of 20 and 60 participated in this prospective cross sectional study. Individuals with LBP were selected among the patients in the orthopedic and physical therapy departments. At first 100 subjects with LBP who were diagnosed with ITB tightness were selected. Then, 100 subjects with LBP without ITB tightness and 100 subjects with no LBP, matched in age and gender to those with ITB tightness, were selected from the same clinical settings as control groups. All the subjects signed an informed consent form approved by the human subjects committee at the University of Social Welfare and Rehabilitation Sciences before participating in the study.

### Selection Criteria

Subjects with LBP were included if they had a history of LBP for more than six weeks prior to the study or had at least three episodes of intermittent low back pain, each one lasting more than one week, during the year prior to the time of the study. Subjects without LBP were included if they had no spinal column pain and had no radicular pain in their lower extremities during one year period before the study. Subjects were excluded if they had history of spinal surgery, spinal or pelvic fracture, hospitalization for trauma of motor vehicle accident, fractures of the lower extremity, hip/knee dysfunctions such as knee valgus/varus, pregnancy, any systemic disease such as arthritis, tuberculosis, liver and/or kidney failure. Subjects with leg length discrepancies, because of its potential effect on ITB length [23] were also excluded. The leg length was measured from the anterior superior iliac spine to the distal medial malleolus with a measuring tape and subjects with leg length difference greater than 10 mm were excluded [20,23].

### Procedure for diagnosing ITB tightness

The Ober test, a common and widely accepted test for measuring the length of the ITB, was used to assess the ITB tightness [9,17,23-25]. This test was performed in the side lying position. Subject's lower leg was flexed at the hip and knee joints. The examiner, standing behind the subject, with one hand, stabilized the pelvis and passively abducted and extended the upper leg with the knee flexed with the other hand. Maintaining extension and neutral position of the hip, the examiner allowed the testing leg to drop toward the table. If subject's leg remained abducted, the subject was considered as having ITB tightness. Based on test results, subjects with LBP were categorized as with or without ITB tightness.

### Measuring hip abductor muscle strength

Hip abductor muscle strength, in this study, was quantitatively measured by a pressure meter similar to the one described by Helewa et al [26,27]. The reliability and validity of this procedure has previously been established [20,26]. The unit used in this study first was calibrated and had 99% measurement accuracy. To measure muscle strength, subjects assumed the standard positions for testing the hip abductor muscle strength [28]. We followed the detail instructions by others [9,17,22] to selected standard contact points to measure the muscles strength. The pelvis was fixed and the inflated bag of the pressure meter was placed between the examiner's hand and the specified contact point for test on the subject's tigh [22]. The pressure meter used in this study provided measurements in kPa units, which is defined as force per unit area. To assure reliability of measurements, hip abductor strength assessments were performed by one therapist. We selected standard contact point, recommended for manual muscle testing, and used the same size inflated bag for all strength measurements. At the end of the test procedure, the subjects were asked if pain was a limiting factor to produce voluntary muscle contraction in assessment of muscle strength. The subjects who had pain during the testing procedure which affect strength testing were excluded from the study. Intra-class correlation analysis revealed ICC (3,1) values equal to 0.92 for reliability of hip abductor muscle strength assessments [20].

### Data Analysis

Subjects who with LBP tested positive on the Ober test were considered as having ITB tightness and those with negative test were classified as having LBP without ITB tightness. Because the effect of Body Mass Index (BMI) and body size on muscle function and strength [29-31], Analysis of Covariance (ANCOVA) with the BMI as the covariate in the analysis was calculated to compare the hip abductor muscle strength across the three groups.

## Results

Descriptive data related to subjects for all three groups is presented in Table 1. There was no statistically significant difference in subjects' age, height, weight and BMI among the three groups. Refer to Table 1 for detailed data.

**Table 1:** Mean Age, Height and Weight of the Subjects in each group.

	With No LBP		LBP with ITBT		LBP with no ITBT		P-values
	**Mean**	**SD**	**Mean**	**SD**	**Mean**	**SD**	

**Age**	43.4	4.41	44.23	13.04	42.58	14.1	0.32

**Weight (Kg)**	70.18	11.45	72.77	11.92	69.10	10.1	0.25

**Height (m)**	1.65	0.09	1.66	0.09	1.66	0.09	0.43

**BMI (Kg/m**^2^**)**	25.68	4.1	26.11	3.34	25.03	3	0.07

LBP = Low Back Pain

ITBT = Iliotibial Band Tightness

Descriptive statistics (Mean, SD) for hip abductor muscle strength in three groups and the results of ANCOVA are provided in Table 2.

**Table 2:** Hip abductor muscle strength for the three groups and ANCOVA with BMI as the covariate.

Variables	With No LBP		LBP with ITBT		LBP with no ITBT		P-values ANCOVA
	**Mean**	**SD**	**Mean**	**SD**	**Mean**	**SD**	

**Hip Abductor Strength (Kpa)**	33.51	7.29	27.07	8.01	27.87	7.95	0 < 001 *

LBP = Low Back Pain

ITBT = Iliotibial Band Tightness

* Post Hoc Analysis: LBP with ITBT vs. LBP without ITBT: P = 0.59;

No LBP vs. LBP with ITBT: P < 0.001;

No LBP vs. LBP without ITBT: P < 0.001

The findings of ANCOVA with the BMI as covariate revealed significant difference in hip abductor strength between three groups (P < 0.001). Post hoc analysis showed that there was no significant difference in hip abductor muscle strength between the LBP subjects with and without ITB tightness (P = 0.59). Subjects with no LBP had significantly stronger hip abductor muscle strength compared to subjects with LBP with ITB tightness (P < 0.001) or those with LBP without ITB tightness (P < 0.001).

## Discussion

The results of this study, in agreement with others [20-22], showed that subjects with LBP, in general, present with weaker hip abductor muscles compared to those without LBP. The results of this study showed that in subjects with LBP, those with ITB tightness had no significantly weaker hip abductor muscle strength compared to individuals without ITB tightness (Table 2).

Considering these findings, it seems that hip abductor muscle weakness is not more pronounced in individuals with LBP with ITB tightness. These findings are in contrast with the notion proposed by others [10,15,16] that ITB tightness could be a compensatory mechanism for providing pelvic lateral stability in subjects with hip abductor weakness.

Some investigators have also hypothesized a common muscle imbalance pattern of weakness in hip abductor and tightness of ITB in chronic LBP [10-15]. It is assumed that when the primary muscle responsible for hip abduction; gluteus medius, is weakened, the synergistic muscle; TFL, is substituted and become overactive to be the primary muscle [10,15,16]. Thus, in theory, it is thought that hip abductor weakness, shown in subjects with LBP, is accompanied with ITB tightness in these subjects. Based on these assumptions, if the proposed theory was true, one would expect a significant difference in the hip abductor strength between subjects with LBP with ITB tightness and those without ITB tightness. In this study, however, no significant difference was found in hip abductor strength between LBP subjects with and without ITB tightness (Table 2). Based on these findings, it seems that ITB tightness might not probably occurred following hip abductor weakness in subjects with LBP as it has been assumed in theory.

The hip abductors help to control rotational alignment of limb and maintain pelvic stability in single leg stance [1,6,7]. It is theorized that weakness of hip abductor muscle may cause a compensatory dynamic valgus knee alignment resulting in increased stress on the ITB. Eggen et al [32] found that knee valgus movement increased after the hip abductors insufficiency. The fact that no significant difference in hip abductor muscle strength was found in subjects with LBP with ITB tightness compared to those without ITB tightness may be due to this that subjects with obvious knee valgus were excluded from this study. Furthermore, although the gluteus medius and ITB are both hip abductors, the gluteus medius is an external rotator of the hip whereas TFL/ITB is an internal rotator of hip. Thus, the function of hip abductor muscle could not be completely substituted by ITB. Similar findings have been reported elsewhere in other musculoskeletal disorders. Sims et al [33] found a significant difference in gluteus medius activation and no significant difference in TFL in subjects with clinical unilateral hip osteoarthiritis compared to a control group. Grau et al [19] in a study of 10 healthy runners and 10 runners with ITB syndrome concluded that hip abductors weakness does not seem to play a role in the etiology of ITB syndrome in runners. It seems that function of muscles and joints in the lower extremity are highly interrelated and weakness or tightness of the muscles might be affected by several factors such as knee, ankle, foot and other disorders [34]. Although no significant difference was found in hip abductor strength between LBP groups with and without ITB tightness, this may be due to the fact that subjects, in this study, were not totally controlled for disorders in other joints in lower extremity.

Another issue should be considered is "pain interference" and intensity level of pain. Some investigators stated that muscle dysfunction in LBP patients might be related to pain, called "pain interference" [35]. They proposed that general ability of voluntary contraction in all muscles might be reduced in patients with LBP because of the pain sensation. Our findings could be criticized because low-level pain might produce the changes the researchers were testing for, whereas those with high pain intensity may have the changes. In this study, the subjects were asked if pain was a limiting factor to produce voluntary muscle contraction in assessment of muscle strength. The subjects who had pain during the testing procedure were excluded from the analysis.

However, one of the limitations of this study was this issue that intensity level of pain was not rated. We wanted to have a more heterogeneous population of patients with chronic LBP with different level of pain.

Another area of concern in our study is that the examiner performing muscle strength test was aware of health status of the participants and ITB tightness. However, the examiner tried to have no bias on strength test results. Cross-sectional studies, including this one, cannot determine the pathophysiology of such association. The relationship between ITB tightness and hip abductor weakness could still be investigated in a longitudinal study.

## Conclusion

In conclusion, the results of this study, in contrast with presented theory, revealed no significant difference in hip abductor strength between subjects with LBP with and without ITB tightness. However, our data indicated that both LBP subjects with ITB tightness and those without ITB tightness have significantly lower hip abductor muscle strength compared with subjects without LBP. It seems that in clinical evidence, ITB tightness might not be due to a compensatory mechanism following hip abductor weakness in subjects with LBP. More clinical studies are needed to assess the stated hypothesis regarding the theory of muscle imbalance between hip abductor muscle weakness and ITB tightness in patients with LBP.

### Clinical implications

The results of this study could be beneficial to clinicians when prescribing therapeutic exercises for patients with ITB tightness, particularly those with LBP.

## Consent/ethics

This research was reviewed and was approved by the Human Subject Committee at University of Social Welfare and Rehabilitation Sciences.

## Abbreviations

ITB: Iliotibial Band; LBP: Low Back Pain; TFL: Tensor Fascia Lata

## Competing interests

The authors declare that they have no competing interests.

## Authors' contributions

Both authors have made substantial contributions to conception and design, acquisition of data, analysis and interpretation of data and have been involved in preparing the manuscript. Both authors read and approved the final manuscript.
